# Prevalence and risk factors for maternal mortality at a tertiary care centre in Eastern Nepal- retrospective cross sectional study

**DOI:** 10.1186/s12884-021-03920-4

**Published:** 2021-07-01

**Authors:** Sarita Sitaula, Tulasa Basnet, Ajay Agrawal, Tara Manandhar, Dipti Das, Prezma Shrestha

**Affiliations:** 1 Department of Obstetrics and Gynecology, BP Koirala Institute of Health Sciences, Dharan, Nepal; 2 Department of Obstetrics and Gynecology, Maharajgunj Medical Campus, TUTH, Kathmandu, Nepal

**Keywords:** Developing countries, Hypertensive disorder of pregnancy, Maternal mortality, Obstetric hemorrhage, Sepsis

## Abstract

**Background:**

The maternal mortality ratio is a significant public health indicator that reflects the quality of health care services. The prevalence is still high in developing countries than in the developed countries.

This study aimed to determine the MMR and identify the various risk factors and causes of maternal mortality.

**Methods:**

This is a retrospective study conducted in a tertiary care center in Eastern Nepal from 16^th^ July 2015 to 15^th^ July 2020. The maternal mortality ratio was calculated per 100,000 live-births over five year’s study period. The causes of death, delays of maternal mortality and, different sociodemographic profiles were analyzed using descriptive statistics.

**Results:**

There was a total of 55,667 deliveries conducted during the study period. The calculated maternal mortality ratio is 129.34 per 100,000 live-births in the year 2015 to 2020. The mean age and gestational age of women with maternal deaths were 24.69 ± 5.99 years and 36.15 ± 4.38 weeks of gestation. Obstetric hemorrhage, hypertensive disorder of pregnancy and sepsis were the leading causes of maternal death. The prime contributory factors were delay in seeking health care and reaching health care facility (type I delay:40.9%).

**Conclusions:**

Despite the availability of comprehensive emergency obstetric care at our center, maternal mortality is still high and almost 75% of deaths were avoidable. The leading contributory factors of maternal mortality are delay in seeking care and delayed referral from other health facilities. The avoidable causes of maternal mortality are preventable through combined safe motherhood strategies, prompt referral, active management of labor and, puerperium.

## Introduction

Maternal mortality is defined as the death of a pregnant women or within 42 days of termination of pregnancy, irrespective of the duration and site of the pregnancy from any cause related to or aggravated by the pregnancy or its management but not from accidental or incidental causes [1]. The maternal mortality ratio (MMR) is a significant public health indicator that determines the quality of health care services and the women’s status in their society [2].

The global maternal mortality ratio showed a significant reduction in maternal deaths (declined by 38 percent) from 342 to 211 deaths per 100,000 live births (from 2007 to 2017), according to UN inter-agency estimates. However, 94% of all maternal death has occurred in low and lower middle income countries. The MMR in low-income countries in 2017 is 462 per 100,000 live births versus 11 per 100,000 live births in high-income countries [3].

The Sustainable Development Goals 3 (SDG 3) plan to reduce the global maternal mortality ratio to fewer than 70 per 100,000 live births by 2030 and have suggested no country should have an MMR greater than 140 per 100,000 live births, a number twice the global target [4].

The Government of Nepal has implemented hospital-based MPDSR (Maternal Perinatal death Surveillance and Response) in 77 hospitals and community- based MPDSR in 12 districts in an attempt to meet the SDG. The Nepal Demographic and Health Survey (NDHS) 2016, have found a decrease in the MMR from 539 to 239 maternal death per 100,000 live births in between 1996 and 2016. About 12% of deaths among women of reproductive age were attributed to maternal deaths [5]. The national census on MMR has not yet been stated after 2016. The data obtained on maternal mortality from DHIS (District Health Information Software) 2 monthly report of HFS (Health and family services) shows MMR of 114 per 100,000 live-births in year 2016–17. The data shows a tremendous reduction in MMR to 53 per 100,000 live- births in year 2019–20.

According to WHO, more than 70% of all maternal deaths are due to hemorrhage, infection, unsafe abortion, hypertensive disorders of pregnancy, and obstructed labor. The major causes of fatalities are poverty; inadequate, inaccessible, and unaffordable health care; low status of women, and illiteracy. The previously conducted studies in Nepal have elucidated hypertensive disorder of pregnancy, obstetric hemorrhage, sepsis, and anemia as the common and preventable causes of MMR. Provision of access to health care and quality care in pregnancy and the puerperium may help to reduce maternal death. Our institute is a tertiary care center located in the Eastern part of Nepal, where almost ten thousand of deliveries occurs in a year. Nearly sixty five percentage of maternal deaths occurred in patients who have sought delayed care or reached health care centers later. Improving antenatal care and identifying high -risk factors at the grassroot level and timely referral to the higher center would decrease the load of maternal mortality.

This study aimed to calculate the MMR and identify its various risk factors. It also aimed to explore the causes of maternal mortality in a tertiary care center of Nepal.

## Materials and methods

### Study setting

This study was conducted at B.P. Koirala Institute of Health Sciences (BPKIHS), is a tertiary care center located in the Eastern part ( province 1) of Nepal. It is situated on the foothills of the lesser Himalayas in the north with its southern tip touching the edge of the Terai region. The hospital provides obstetric and gynecological services to the women of province number one and two as well as some parts of India (Bihar and Uttar Pradesh). Annually out of 10,000 to 12,000 deliveries conducted in the hospital, only around 30- 40% of women seeking delivery services are registered or booked to the hospital. The rest are unregistered but directly coming to the hospital or referred by other health centers. Most of the unregistered cases present to us at the term period of gestation for the delivery with few or no antenatal visit.

NDHS 2016, projects more than 84% of women has received antenatal care (ANC) from a skilled provider (doctor, nurse, and auxiliary nurse midwife). More than half (57%) have delivered in a health facility, primarily in the government sector facilities and 41% of births are delivered at home without medical supervision.

The annual report of Nepal (2018/19) shows 61% of women had at least 4 ANC checkups as per protocol and birth attended by SBA (skilled birth attendant), 62% had an institutional deliveries where 30% were delivered by cesarean section in province 1.

The government of Nepal has endorsed safe motherhood program to ensure free service and encourage women for institutional delivery has improved access to institutional deliveries and emergency obstetric care services. There is also provision of incentives to those who delivers at a hospital.

The geographical variation and lack of efficient transport system pose great difficulties in seeking appropriate timely care. Financial constraints are another bearing factor for the underprivileged in our part of the world.

### Study design

This was a retrospective descriptive study of five years. Data were collected from the files from the record section. Detailed retrospective reviews of records were undertaken from the case notes of 56,787 admissions in the obstetrics unit during 5 years period from 16^th^ July 2015 to 15^th^ July 2020. The information was retrieved from the case notes available in the medical records department of the hospital as well as from the daily records in the maternity and delivery sections of the hospital during the study period.

The study population included women who delivered in the hospital as well as referred from other centers presenting with pregnancy-related complications and expired during management. The study included the mortalities due to ectopic pregnancy, molar pregnancy, abortion, and its related complications. The study excluded the case of “dead on arrival” during pregnancy or puerperium as details of pregnancy or required data would not be available or identified. The death of pregnant/ puerperal women due to violence or accidental cause was also excluded, as these cases are not under the definition of maternal death.

Data retrieved from case notes included general socio-demographic profile, clinical presentation, history of present and past obstetric outcomes, primary and final cause of death. Total number of deliveries, total number of live births, and number of maternal mortality during this period were noted. Additional methods used to identify the number of maternal mortalities in the hospital included review of daily delivery records, review of records in the surgical departments as well as records from maternal death reviews which are conducted periodically in the hospital.

The study was conducted after ethical clearance from the Institutional Review Committee (IRC number: IRC/1772/020) of the hospital. The Hospital Director and the Head of the Department of the hospital were informed about the purpose of the study, and approval was obtained for the retrieval of data from case sheets. The confidentiality of information obtained is assured. Informed consent from the patient to review their case sheets was waived due to retrospective and non-interventional study design.

### Study variables

Demographic characteristics: age, gravida, residence and ethnicity.

Obstetric characteristics: antenatal care, booking status, presentation to the hospital(antenatal/ postpartum), condition of women at presentation, complications during the index pregnancy, POG at delivery, mode of delivery, place of delivery and complications during delivery.

Variables related to maternal mortality: Maternal mortality ratio, cause of mortality (primary cause and contributory cause), the time duration from presentation to mortality, the timing of mortality (early pregnancy, antepartum, postpartum) and types of delay.

### Data collection tool

The data were collected from the maternal death review (MDR) form designed by the Government of Nepal, Family Health Division.

### Statistical analysis

The data were entered in a Microsoft Excel sheet and analysis was performed using SPSS version 24. Descriptive statistics like frequency, percentage, mean and standard deviation were calculated and presented in tables.

The characteristics of the study population and prevalence of maternal death were presented using percentage and absolute numbers. MMR was calculated as the number of deaths due to maternal causes per 100,000 liver births within the period.

### Definition of the study variable

#### Maternal mortality ratio

The number of maternal deaths per 100,000 live births in a given year and area.

#### Booking status

Booked: At least one antenatal visit to the hospital. Unbooked: no antenatal checkup at the hospital.

#### Timing of mortality

Death during Early pregnancy- Death occurred in the early trimester which is related to ectopic pregnancy, molar pregnancy, or complications related to abortion.

#### Cause of mortality

Primary cause: The disease/ event which initiated the chain of events leading to death.

Contributory cause: Condition that may exist prior to development of the primary cause of death or developed during the chain of events leading to death. Eg cardiac disease.

Final cause: Final disease or condition or complication leading to death. Eg hypovolemic shock.

#### Types of delay:

- Delay 1- Delay in deciding to seek care.
- Delay 2-Delay in reaching the healthcare facility.
- Delay 3- Delay in receiving care.

## Results

There was a total of 55,667 deliveries and 54,892 live births over the study duration of five years. During this period, there were 71 maternal deaths and the calculated maternal mortality ratio (MMR) was 129.34 per 100,000 live births.

**Fig. 1:**
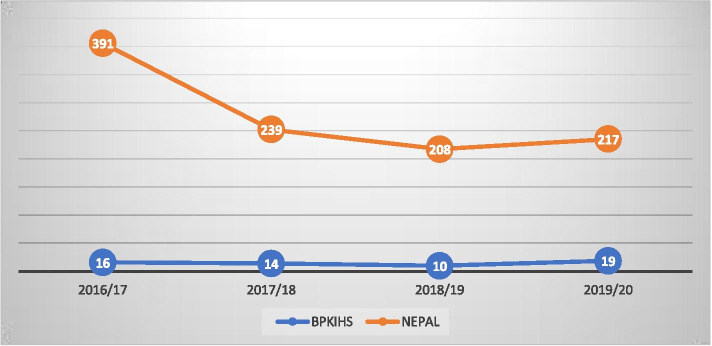
Number of maternal deaths during four years

Among 71 maternal death, 65 women had death related to late obstetric events and six deaths were related to early pregnancy complications (abortion, molar, and ectopic pregnancy). They are analyzed in detail separately. Figure 1 shows the yearly number of maternal deaths in the hospital in comparison to the maternal deaths of whole country.

The baseline characteristics of all maternal mortality are presented in Table 1. The most common age group of the women with maternal mortality ranged from 20 to 34 years with a mean age of 24.69 ± 5.99. The most common ethnic group was Brahmin (31%) followed by Janajati (25.4%). Seventy percentage of women presented in the emergency room in a state of shock (mostly hypovolemic shock) and 60% required blood transfusion at or after admission.

**Table 1:** Maternal mortality and its characteristics (*n* = 71)

Characteristics	Frequency (%)
Age (years)	
≤ 19	10 (14.1)
20–34	55 (77.5)
≥ 35	6 (8.5)
Gravida	
1	31 (43.7)
2–4	34 (47.9)
≥ 5	6 (8.5)
Residence	
Mountain	3 (4.2)
Hill	11 (15.5)
Terai	57 (80.3)
Shock at admission	
Yes	21 (29.6)
No	50 (70.4)
Referral	
Referred	49 (69.0)
Not referred	22 (31.0)
ICU admission	
Yes	58 (81.7)
No	13 (18.3)
Admission to death duration	
< 24 h	23 (32.4)
> 24 h	48 (67.6)

The duration of hospital stay (admission to death time) was less than 24 h in around 32%. Eighty two percentage of women required maternal ICU for critical care management or ventilatory support. Other women (18%) had died in the emergency itself during the resuscitation process as they had arrived in a crucial state.

Table 2 outlines the obstetric characteristics of women who had mortality during antenatal or in the postpartum period.

**Table 2:** Characteristics of obstetric case among maternal mortality (*n* = 65)

Characteristics	Frequency (%)/ Mean ± SD
Period of admission	
Antepartum	43 (66.2)
Postpartum	22 (33.8)
Period of death	
Antepartum	18 (27.7)
Postpartum	47 (72.3)
Gestational age(weeks)	36.15 ± 4.38
ANC care	
Yes	56 (78.9)
No	9 (12.7)
Don’t know	6 (8.5)
Booking status	
Booked	3 (4.6)
Not booked	62 (95.4)
Complication in index pregnancy (*n* = 65)	
Yes	47 (72.3)
No	18 (27.7)
Mode of delivery	
Vaginal	24 (36.9)
Cesarean	23 (35.4)
Not delivered	18 (27.7)
Place of delivery (*n* = 47)	
BPKIHS	28 (59.6)
Outside	19 (40.4)
Fetal outcome (*n* = 47)^a^	
Alive	32 (68.1)
Stillbirth	12 (25.5)
NND	2 (4.3)
Hysterectomy	
Yes	8 (12.3)
No	57 (87.7)

^a^one women had spontaneously expelled fetus at 19 weeks

Among 65 women 22 of them were admitted during the postpartum period and almost 45% (10 case) were admitted during first 48 h of delivery with postpartum hemorrhage in a state of shock. Though 79% of women had received at least a few visits to antenatal care, only around 4.6% had done antenatal checkups in our hospital. After arrival at our center, 18 women (27%) had died prior to delivery of the fetus during the antepartum or intrapartum period.

**Fig. 2:**
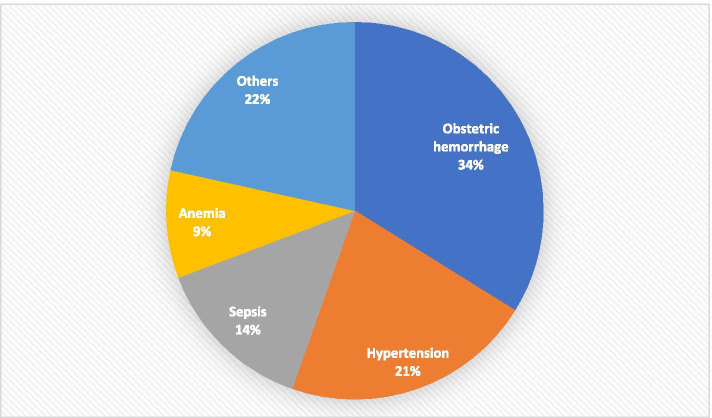
Primary causes of maternal mortality (*n* = 65)

We had also searched for the primary cause of maternal mortality and is as shown in Fig. 2. The study has produced overwhelming evidence to state that obstetric hemorrhage comprising 34% of the cases is still the leading cause of death in our setup. Out of 22 cases of obstetric hemorrhage, 17 cases had a primary postpartum hemorrhage, and 5 had presented with antepartum hemorrhage(APH) i.e., three with placenta previa and two with placental abruption. Eight cases (8/22) underwent peripartum hysterectomy of which 3 cases were associated with morbidly adherent placenta and five for atonic PPH. The final cause of death was hypovolemic shock (*n* = 10), multiple organ dysfunction syndrome (*n* = 11), and heart failure in one case who had pre-existing heart disease.

The second most common cause of death was related to the hypertensive disorder of pregnancy. Among 14 cases with hypertension, eight cases had presented with eclampsia at the time of admission. The final cause of death was pulmonary edema(*n* = 6), multiple organ dysfunction syndrome (*n* = 5), Cardiovascular accident (*n* = 1) and aspiration pneumonia (*n* = 2).

Fourteen percent of women had died due to sepsis emphasizing puerperal sepsis as the third common cause of maternal mortality. The denominator causes of sepsis were surgical site infection (*n* = 2), urosepsis (*n* = 2), and acute respiratory distress syndrome due to pneumonia (*n* = 4). Almost all women with sepsis had died of septic shock. The primary cause of death in 9% of women was related to anemia and its complication.

The other causes of death were related to uterine rupture (*n* = 3), heart disease (*n* = 3), acute fatty liver of pregnancy (*n* = 1), suspected pulmonary embolism (*n* = 3), and SLE (*n* = 1).

Six women had mortality related to early trimester complications which are enlisted in Table 3.

**Table 3:** Details of women who had mortality related to molar, ectopic and abortion (*n* = 6)

Cause of death	Frequency
Ruptured ectopic pregnancy with hypovolemic shock	1
Complications of molar pregnancy	2
Unsafe abortion leading to septic abortion	3

Assessment of types of delays associated with maternal mortality showed that the majority of deaths were associated with type I delay (40.9%) and were reported to be due to delay in seeking health care or due to delay in reaching health care facility. Type II delay was seen among 23.9%, which were due to late referral from other facilities. Likewise, type III delay was seen in 21.1% of deaths as explained in details in Table 4.

**Table 4:** Distribution of maternal mortality according to three types of delays

Types of delay	Frequency (%)
Delay 1	29 (40.9)
Delay 2	17 (23.9)
Delay 3	15 (21.1)
Lack of supplies/ equipment	6
Inadequate skill of provider Delay in receiving	2
treatment in the health facility	7
No delay	10 (14.1)

## Discussion

Deaths from complications of pregnancy and childbirth are high with WHO recording 295,000 maternal deaths globally in 2017 [2].

This study was conducted to identify the maternal mortality ratio and causes of maternal death in a tertiary care center in a developing country. The MMR was found to be 129.34 per 100,000 live births, which is lesser as compared to other reviews done in developing countries [6, 7]. The MMR is very much less in our center as compared to the rate as mentioned by NDHS 2016 (MMR of 239) as this is a hospital based study [5].The common causes of maternal mortality in our center were obstetric hemorrhage, hypertension, sepsis, and anemia which is similar to the findings from other studies [7–10].

An observational study conducted in a tertiary care referral center of Western Nepal found that MMR of 151 per 100,000 live births with the mean age of the mother being 28 years. Most of the patients had presented to the center in unstable health conditions, with a common cause of death being hypertension and sepsis. These findings were also comparable to our study. Most of them (73.30%) had died in the postpartum period [11].

In this study, almost 70% of women were referred from other health care centers. Almost 30% of cases presented in a state of shock at the time of admission resulting in delayed intervention and hence adverse outcomes. It was similar to the findings from other studies done in developing countries [7, 12, 13].

In a study conducted in Nigeria, six leading causes of maternal mortality were hemorrhage, eclampsia/ preeclampsia, sepsis, ruptured uterus, complications of abortion, and prolonged obstructed labor. Among these causes, 43.4% accounted for hemorrhage followed by 36.0% of preeclampsia and eclampsia which coincides with the finding seen in our study [12]. In our study 3, women had ruptured uterus. Among them, 2 women were referred from outside after delivery in a state of shock and expired during the resuscitation process. Other women had ruptured uterus diagnosed during the intrapartum period. The women who were referred from outside had a difficult vaginal delivery and gave history of fundal pressure. It seems that the use of fundal pressure during vaginal delivery is still being practiced in peripheral setup.

Another study conducted in India, had found MMR of 802 per 100,000 live births which was very much higher than the finding of our study. In this study, maternal anemia (53.57%) was the most common morbidity unlike the finding in our study where hypertensive disorder of pregnancy was the most common comorbidity. Almost 93% of death had occurred in the postpartum period and 94.6% of women were referred from another center [7].

Overall, high MMR was found in various studies which were conducted in a referral centers in developing countries, which reported MMR of 426 per 100,000 live births and 1513.4 per 100,000 live births [14]. Comparable to several studies, most of the death (77.50%) had occurred in women of 20- 34 years of age [7, 8, 10, 15]. The mean gestational age at death is 36.15 ± 4.38 weeks in our study which is similar to another study [10].

Evidence has suggested that three delays are important factors for maternal morbidity and mortality in Nepal. The delay I (40.9%) was seen in maximum death followed by delay II in our study. Most of these cases were related to late referrals from other health centers. This calls for strengthening the capacity of the health care workers in early recognition of danger signs and referral to the appropriate centers on time.

This study has highlighted the gaps between the community to the tertiary care center. Those women who had delivered at home or primary care center are being referred to many other centers before reaching tertiary care center or not referred on time due to lack of skills/ knowledge to identify the high-risk patient. Furthermore, delays in interventions and inadequate supply of equipment, inadequate skills of providers had also contributed to the deaths.

The following significant strategies have been adopted to reduce risk during pregnancy and childbirth and address factors associated with mortality and morbidity at national level:

Promoting birth preparedness and complication readiness including awareness- raising and improving preparedness for funds, transport and blood transfusion.

Expansion of 24 h birthing facilities alongside a safe motherhood program, which also promotes the continuum of care from antenatal care (ANC) to post-natal care (PNC). The expansion of 24 -hour emergency obstetric care services (basic and comprehensive) at selected health facilities in all districts.

The steps taken by government are commendable. However, there is unequal distribution of health care services throughout the country based upon population distribution. Also, only selected health facilities give BEONC and CEONC, easy access to those facilities is far from reality due to difficult transport system especially in hilly and mountainous areas. Having said so, more than half of our study population were from Terai. This may be because our hospital is in vicinity of Terai region. In addition to that, even though the transport in Terai region is easy, the awareness regarding reproductive health care, right of women on deciding about their own health is lacking. Also, because of patriarchal society, there is tendency of multiparity till a woman delivers a son. Another aspect responsible for increased maternal mortality is unavailability of trained health care workers in all facilities. Though the provision of easy transport and social reform will take time, providing training to the health care workers to increase their skills in prevention, detection, and management of the leading causes of maternal deaths through an obstetric drill, refresher course may help in reducing maternal morbidity and mortality to some extent. In our study, few deaths were related to delay in providing intervention, inadequate supply of the instruments (delay 3) which need to be addressed by the hospital management in coordination with the province and national government.

The government can also plan on establishing a confidential maternal death enquiry (CMDE) which is already started in many countries like England and Wales, Malaysia, Ireland. The aim of CMDE is not just to ascertain the numbers of deaths but principally to promote safer pregnancy by learning how such tragedies could be avoided in the future. This could make a major contribution to informing and improving standards of care in maternity services and the use of guidelines and recommendations would help to ensure all the pregnant and recently delivered women receive the best possible care.

Our institute routinely conducts a maternal-perinatal death audit, a surgical audit and identifies the lacunae in management that has led to the mortality. Such audits are helping us to formulate short term and long term plans to act properly if such conditions arise in future and to reduce MMR.

### Strengths and limitations

This hospital-based study provides little representation of what is happening in the community and may lead to under-reporting. However, this study done over 5 years of duration provides trends of maternal mortality in our population. Also, this sample may not represent the general population as this is a referral center where patients were self-referred or referred by another center. As this is a descriptive study that lack a comparison group, it may not provide a causative association for maternal deaths. The contributory factors leading to delay I and II were also not studied separately.

As this is a retrospective study with less sample size, a more extended study period with a large sample size would give meaningful data. However, we have ensured accurate data using multiple approaches to identify all cases of maternal deaths in the hospital.

## Conclusions

Despite the availability of comprehensive emergency obstetric care at our center (BPKIHS) maternal mortality is still high and almost 75% of deaths were avoidable. The leading contributory factors were due to delay in seeking care or delayed referral from other health facilities. Contributory factors related to maternal mortality are preventable through combined safe motherhood strategies, prompt referral, active management of labor, and puerperium. The findings of this study call for improving/ identifying high-risk patients on time with a timely referral with proper documentation so that early action/ intervention can be done in a referring center in case of an obstetric emergency. We require more studies to address all the delays of maternal mortality and find its causative association.

## Data Availability

the datasets used and / or analyzed during the current study are available from the corresponding author on reasonable request.

## Abbreviations

ANC: Antenatal Checkups
APH: Antepartum Hemorrhage
CMDE: Confidential Maternal Death Enquiry
ICU: Intensive Care Unit
MMR: Maternal Mortality Ratio
MPDSR: Maternal Perinatal Death Surveillance and response
PNC: Postnatal Checkups
POG: Period of Gestation
PPH: Postpartum Hemorrhage
NDHS: Nepal Demographic and Health Survey
SDG: Sustainable Development Goals
